# Early post-treatment with 9-cis retinoic acid reduces neurodegeneration of dopaminergic neurons in a rat model of Parkinson’s disease

**DOI:** 10.1186/1471-2202-13-120

**Published:** 2012-10-06

**Authors:** Lian-Hu Yin, Hui Shen, Oscar Diaz-Ruiz, Cristina M Bäckman, Eunkyung Bae, Seong-Jin Yu, Yun Wang

**Affiliations:** 1 Neural Protection and Regeneration Section, Intramural Research Program, National Institute on Drug Abuse, Baltimore, MD, USA; 2 Integrative Neuroscience Section, National Institute on Drug Abuse, IRP, Baltimore, MD, USA; 3 Department of Neurosurgery, Luhe Teaching Hospital, Capital Medical University, Beijing, China

## Abstract

**Background:**

Retinoic acid (RA) is a biologically active derivative of vitamin A. Previous studies have demonstrated that RA has protective effects against damage caused by H_2_O_2_ or oxygen-glucose deprivation in mesangial and PC12 cells. Pretreatment with 9-cis-retinoic acid (9cRA) reduced infarction and TUNEL labeling in cerebral cortex as well as attenuated neurological deficits after distal middle cerebral artery occlusion in rats. The purpose of this study was to examine a protective role of 9cRA in dopaminergic (DA) neurons in a typical rodent model of Parkinson’s disease (PD).

**Results:**

The protective role of 9cRA was first examined in rat primary ventromesencephalic culture. Treatment with 9cRA significantly reduced 6-hydroxydopamine (6-OHDA)-mediated cell death and TUNEL labeling in cultured dopaminergic neurons. The protective effect was also examined in adult male rats. Animals received unilateral 6-OHDA lesioning at the left medial forebrain bundle on day 0. Methamphetamine -induced rotational behavior was examined on days 6, 20 and 30 after lesioning. Animals were separated into 2 groups to balance rotational behavior and lesion extent on day 6 and were treated with either 9cRA or vehicle (i.c.v. on day 7 + intra-nasal from day 8 to day 14). Post-treatment with 9cRA significantly reduced methamphetamine –mediated ipislateral rotation at 20 and 30 days after lesioning. *In vivo* voltammetry was used to examine DA overflow in striatum. Treatment with 9cRA significantly increased KCl -evoked DA release in the lesioned striatum. 9cRA also increased tyrosine hydroxylase (+) cell number in the lesioned nigra as determined by unbiased stereology.

**Conclusion:**

Our data suggests that early post-treatment with 9cRA has a protective effect against neurodegeneration in nigrostriatal DA neurons in an animal model of PD.

## Background

Retinoic acid (RA) is a biologically active derivative of vitamin A. Two major isoforms of RA, all-trans RA (atRA) and 9-cis RA (9cRA), have been identified. atRA is normally present at high levels in the developing spinal cord and at low levels in the forebrain of mouse embryos (Horton and Maden, 1995). On the other hand, 9cRA is not present in mouse embryos [1-3] or tissue extracts from adult rats [4]. RAs interact with two major groups of nuclear receptors: retinoic acid receptors (RAR) and retinoid X receptors (RXR). 9cRA binds with high affinity to RXR [5], whereas both 9cRA and atRA activate RAR [6,7].

RA has been reported to have protective effects after injury. RA inhibited H_2_O_2_-induced apoptosis via suppression of c-fos/c-jun expression and JNK activation in mesangial cells [8] and increased survival during anoxia/glucose deprivation in PC12 cells [9]. These data suggest that RA can induce protective responses in cultured cells. The function of RA in the mature nervous system is less documented. Pretreatment with docosahexaenoic acid (DHA), a candidate ligand for RXR [10], reduced cerebral infarction induced by middle cerebral artery occlusion [11,12]. We previously reported that 9cRA is more potent than atRA in reducing ischemia –mediated brain infarction [13] and, furthermore, pretreatment with 9cRA attenuated DNA fragmentation in the lesioned cerebral cortex of stroke rats [14]. Taken together, these data suggest that 9cRA is neuroprotective against cerebral ischemia *in vivo*.

Several reports also indicate that RA can reduce degeneration induced by dopaminergic neurotoxins in culture. RA attenuates 6-hydroxydopamine (6-OHDA) and 1-methyl-4-phenylpyridinium (MPP+) -mediated neurotoxicity in SY5Y neuroblastoma cells [15]. The protective effects of RA in these cells involves the Akt pathway, increasing BCL2 while reducing p53 levels [15,16]. In rat midbrain slice cultures, treatment with a RAR agonist AM80 prevents IFN-r/LPS –induced dopaminergic cell loss [17]. These data suggest that RA has protective effects in dopaminergic neurons in culture. One recent study has shown that systemic administration of a high dose of RA (1mg/kg, i.p.) for 15 days reversed rotenone –induced impairment of locomotor activity without significantly reversing the reduction of dopamine (DA) in striatum [18]. In most of these studies, atRA was used to examine the protective effect of RA against dopaminergic neurotoxins. The protective role of 9cRA in dopaminergic neurons in an established model of PD has not been reported.

9cRA has also been shown to induce protection in animal model of stroke [13,14]. Application of a similar dose of atRA did not reduce the size of infarction after middle cerebral artery occlusion in rats, suggesting that 9cRA is more potent than atRA in neuroprotection [13]. Unlike atRA, 9cRA regulates the expression of proteins in the transforming growth factor (TGF) -β superfamily. 9cRA enhances production of glial cell line derived neurotrophic factor (GDNF), and bone morphogenetic protein-7 (BMP-7) protein [14], in human neuroblastoma cells and U2 OS cells, respectively. 9cRA also increases midkin mRNA in primary cortical neurons [13]. All these trophic factors or proteins have been reported to reduce stroke insults [13,19,20].

We previously demonstrated that intracerebroventricular administration of 9cRA time-dependently upregulates BMP7 mRNA expression in rodent brain [14]. The 9cRA –mediated reduction in cerebral infarction in stroke rats was significantly antagonized by the BMP antagonist noggin [14], suggesting that 9cRA induces protection through BMP7. Interestingly, BMP7 has been shown to reduce dopaminergic neurotoxins –mediated neurodegeneration *in vivo* and *in vitro*. BMP7 significantly reduced rotational behavior, partially preserved KCl- induced DA release in the lesioned striatum, and increased tyrosine hydroxylase (TH) immunoreactivity in lesioned nigra in unilaterally 6-OHDA -lesioned rats [21]. Similarly, BMP7 antagonized high dose methamphetamine –induced DA cell loss in cultured dopaminergic neurons and striatal TH fiber density reduction *in vivo*[22]. Knocking down BMP7 or BMPRII receptors expression in mice increased sensitivity to dopaminergic neurotoxins in nigrostriatal dopaminergic neurons [22,23]. These data suggest that BMP7 has protective effects against DA-ergic neurotoxin –mediated degeneration. Since 9cRA enhances BMP7 expression, we reasoned it may also induce protection against 6-OHDA lesioning in an animal model of Parkinson’s disease (PD) through the BMP7 pathway.

In this study, we examined the protective effect of 9cRA in a rodent animal model of PD. 9cRA was given at low dose from 7 days after unilateral 6-OHDA lesioning in adult rats. We found that post-treatment with 9cRA significantly reduced rotational behavior while increased the survival of dopamine cells in the lesioned nigra and KCl-evoked DA release in lesioned striatum. Our data suggest that 9cRA induces neuroprotection against neurodegeneration in nigrostriatal DA neurons in an animal model of PD.

## Results

### VM cell cultures

**Figure 1:**
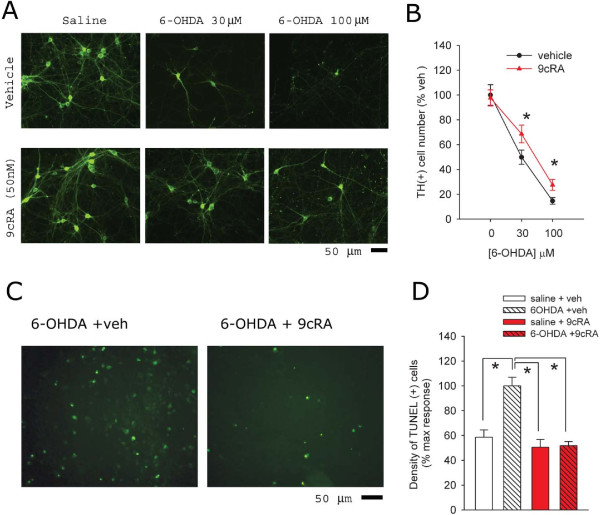
**9cRA reduces 6-OHDA-induced toxicity in ventral mesencephalic cultures. **(**A**) Primary cultures prepared from rat VM (E15) were treated with 6-OHDA (0, 30, 100 μM) for two hours then washed and exposed to vehicle or 9cRA for 22 hours. Cells were fixed and assayed using TH immunostaining. 9cRA (50 nM) alone did not alter the TH (+) neurons in culture; however, it antagonized the 6-OHDA-mediated decrease in TH cell density. (**B**) A dose-dependent effect of 6-OHDA on loss of TH cells. Treatment with 9cRA (50 nM) significantly antagonized the loss TH cells induced by 6-OHDA (*p<0.05, 2- way ANOVA). The density of TH (+) cells was normalized to the cell density in the non-6-OHDA and non-9cRA (vehicle) -treated group. (**C**) 9cRA reduced 6-OHDA (100 μM) –mediated TUNEL labeling in VM neuronal culture. (**D**) Averaged TUNEL density per field. All data were normalized to the mean of TUNEL density in 6-OHDA +veh group. Treatment with 9cRA (50 nM) significantly reduced 6-OHDA- mediated TUNEL activity to control levels (*p<0.001, 1- way ANOVA).

We first examined protective effects of 9cRA in ventromesenphalic (VM) neuronal culture. Cells were fixed at 24 hours after 6-OHDA treatment. Typical TH immunoreactivity of VM cultured cells is demonstrated in Figure 1A. Treatment with 6-OHDA dose-dependently reduced TH (+) cell density (Figure 1B, p<0.001, two way ANOVA). 9cRA or vehicle was given at 2 hours after addition of 6-OHDA or vehicle to the well. Treatment with 9cRA (50 nM) did not alter TH cell density in non-lesioned cells; however, 9cRA significantly attenuated 6-OHDA-medated loss of TH cells at 24 hours after lesioning (Figure 1B, p=0.022, two way ANOVA). We also found that 6-OHDA treatment enhanced the density of TUNEL labeling. 9cRA significantly reduced 6-OHDA –mediated TUNEL activity (Figure 1C and D, p<0.001, one way ANOVA + Newman-Keuls test).

**Figure 2:**
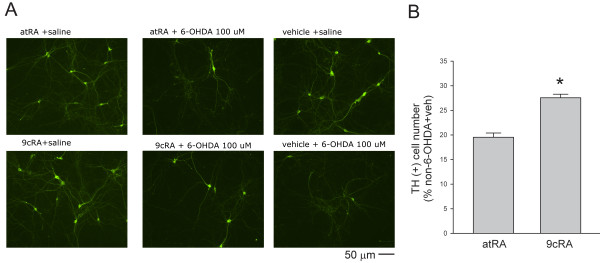
**9cRA is more effective than atRA against 6-OHDA –mediated neurodegeneration in VM TH neurons. **(**A**) Primary VM neuronal cultures were treated with either 6-OHDA (100 μM) or saline for two hours and then were treated with either 9cRA or atRA (50 nM). 9cRA, compared to atRA, is more potent in protecting against 6-OHDA –mediated loss of TH cells). (**B**) 9cRA (50 nM) is significantly effective than atRA (50 nM) to increase survival of TH cells after lesioning with 6-OHDA (100 μM). The density of TH (+) cells was normalized to the cell density in the non-6-OHDA and non-9cRA (vehicle) -treated group. More TH neurons survived after treatment with 9cRA+ 6-OHDA, compared to atRA + 6-OHDA (*p<0.001).

We previously demonstrated that 9cRA is more potent than atRA against ischemic brain injury *in vivo*. Similarly, we found that 9cRA is more efficient that atRA against 6-OHDA –mediated neurodegeneration in VM TH neurons. More TH neurons survived post-treated with 9cRA, compared to atRA, after 6-OHDA lesioning (Figure 2, p<0.001, t test).

### Animal study

#### (A) Rotation

**Figure 3:**
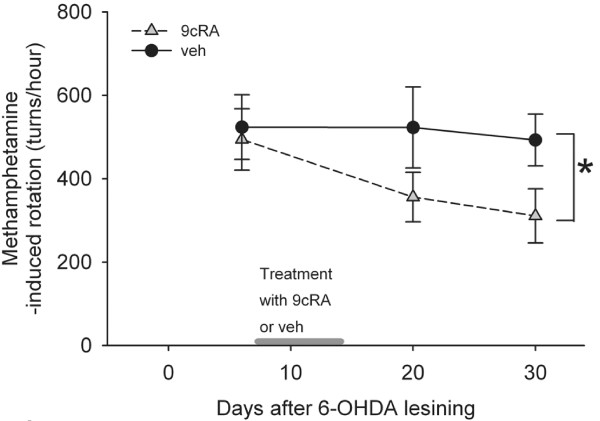
**Post-treatment with 9cRA reduced methamphetamine –induced ipislateral rotation in hemiparkinsonian rats. **Animals were lesioned by local administration of 6-OHDA at the left medial forebrain bundle on day 0. Rotation was induced by administration of 2.5 mg/kg methamphetamine at day 6 (prior treatment) and days 20 and 30 (after treatment). Treatment with 9cRA from day 7 to day 14 (i.c.v. and intranasal) significantly reduced rotation. Bar on X-axis represents the period animals received 9cRA or vehicle.

A total of 17 rats received unilateral 6-OHDA lesioning on day 0. Methamphetamine -induced rotational behavior was examined on days 6, 20 and 30 after 6-OHDA lesioning. Animals were separated into 2 groups (veh and 9cRA) to equalize group rotational behavior and thus lesion extent on day 6. Two animals were excluded because the rotation was higher than 960 turns/hour to avoid maximal lesioning before neural protective treatment. There was no difference in rotation prior to 9cRA or vehicle treatment between these two groups (p=0.789, t test). Averaged rotation (turns/hour) was 494.1 +/− 73.6 (for RA group, n=7) and 523.6 +/− 77.6 (for veh group, n=8). Animals were treated with 9cRA or veh from day 7 to day 14 (Figure 3). The rotational behavior was re-examined on days 20 and 30 after lesioning (Figure 3). Using a two way ANOVA, we determined that 9cRA, compared to vehicle, significantly reduced methamphetamine -mediated rotation in the unilaterally 6-OHDA-lesioned rats (p=0.028, Figure 3).

#### (B) KCl-induced DA release and DA clearance in striatum

**Figure 4:**
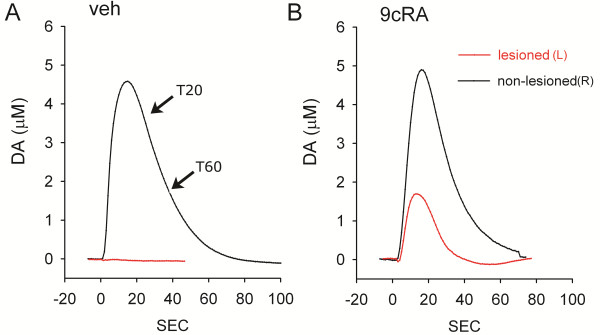
**Typical voltammetric tracings of extracelluar DA concentration in dorsal striatum in unilaterally 6-OHDA lesioned rats. **Examples of KCl -evoked dopamine release were taken from dorsal striatum (3.5 to 5 mm below brain surface). KCl (150–200 nL x 70 mM) was delivered locally through a micropipette next to DA sensor at time 0. (**A**) Local administration of KCl induced DA release in non-lesioned striatum (black tracing) in a vehicle treated animal. In the same animal, KCl-mediated DA release was almost abolished in lesioned striatum (red tracing). (**B**) In contrast, 9cRA treatment partially restored KCl –induced DA release in lesioned striatum in another animal. 9cRA did not alter DA release in the non-lesioned striatum. Note that slope between T20 and T60 (**A**) is used to calculate the rate of clearance in Figure 5B.

KCl-evoked DA release in striatum was examined using *in vivo* voltammetry in 11 rats at >2 months after unilateral 6-OHDA injection. Five of these animals were treated with 9cRA and the other six were treated with vehicle. KCl-evoked DA release was recorded in 143 striatal sites between 3.5 mm to 7.0 mm below the brain surface and 2.0-2.5 mm lateral, 0–1 mm anterior to bregma. Of these, 80 sites were taken from the striatum ipsilateral (L) to the 6-OHDA injection while 44 sites were recorded in the contralateral (R) striatum. Average dose of KCl ejected from micropipette was 204.0 ± 9.1 nl per site. Local application of KCl resulted in release of dopamine in all sites in non-lesioned (R) striatum in both 9cRA and vehicle -treated mice. On the other hand, the peak of DA release was reduced in the lesioned striatum. Typical extracellular dopamine tracings from left and right striatum are shown in Figure 4.

**Figure 5:**
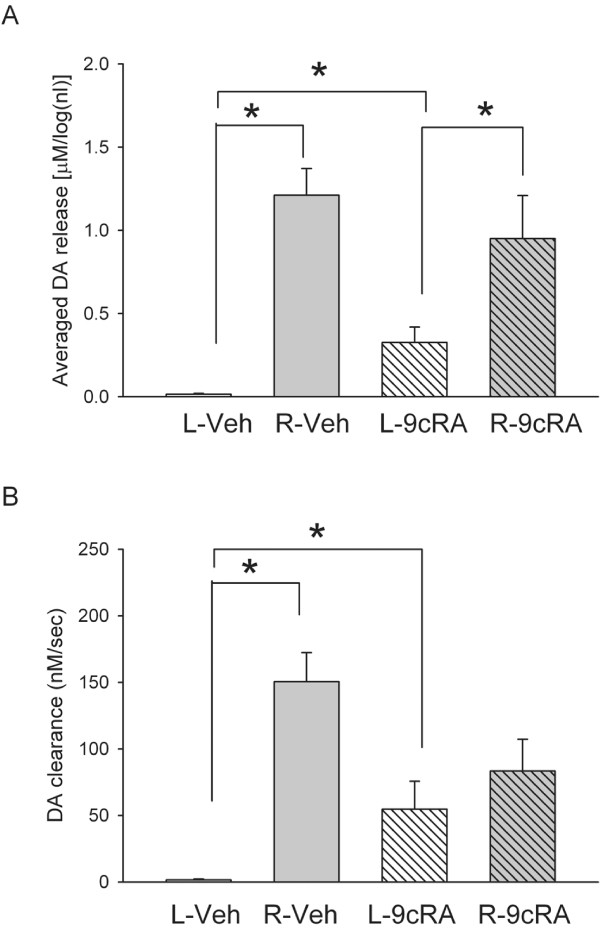
**Averaged DA release (A) and rate of DA clearance (B) in striatum in 9cRA or vehicle treated hemiparkinsonian rats. **(**A**) The peak amplitude of the DA signal, induced by local KCl administration, was averaged in lesioned (L, left) or non-lesioned (R, right) striatum of unilaterally 6-OHDA -lesioned rats. KCl-evoked DA release was almost abolished in the L striatum in vehicle treated rats. Treatment with 9cRA significantly increased DA release in the lesioned striatum. (**B**) The rate of DA clearance (nM/s) after KCl application was calculated between T20 and T60. There is a significant reduction in DA clearance in the striatum ipsilateral to the 6-OHDA lesioning in vehicle treated rats. 9cRA treatment significantly enhanced rate of DA clearance.

Previous voltammetric studies have shown a dose response relationship between the peak of extracellular DA level and log dose of applied DA through a micropipette in rat striatum [24,25]. In this study, the amplitude of DA release was thus normalized by comparison to the log volume (in nL) of KCl used. A similar approach has been used in our previous papers [26,27]. There is a significant reduction in KCl-evoked DA release in the lesioned striatum, compared to the non-lesioned striatum (Figure 5, p<0.001, one way ANOVA; p<0.001) in both vehicle and 9cRA treated animals (p<0.001, post-hoc Newman Keuls test). However, treatment with 9cRA significantly increased KCl-evoked DA release in the lesioned side striatum (p=0.041; Figure 5A), compared to vehicle. No difference was found in the non-lesioned side striatum between 9cRA and vehicle –treated animals (p=0.263) .

The rate of DA clearance (nM per sec) after KCl application was calculated by measuring the slope between T20 and T60 (Figure 4A) of the DA currents, as described previously [27,28]. There was a significant reduction of DA clearance in the lesioned side striatum (Figure 5B, p<0.001, one way ANOVA; p<0.001, post-hoc Fisher LSD test). Treatment with 9cRA significantly increased DA clearance rate in the lesioned side striatum (p=0.028; Figure 5B) compared to vehicle.

#### (C) TH immunoreactivity in nigra

**Figure 6:**
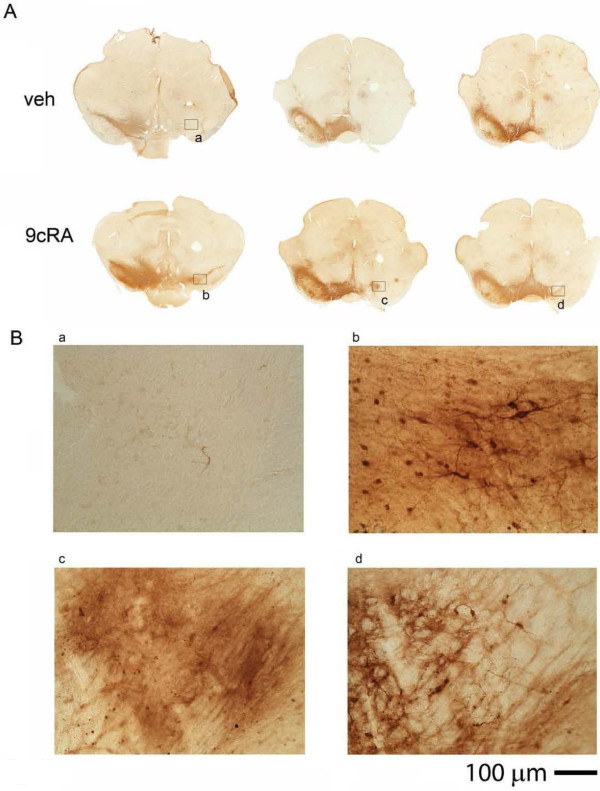
**Post-treatment with 9cRA attenuated the loss of TH cells in nigra. **TH immunoreactivity was conducted at >2 months after unilateral 6-OHDA lesioning. (**A**) Almost no TH activity was found in the lesioned side nigra (left panel, rostral nigra; middle and right panels, caudal nigra) in 3 vehicle treated animals. Treatment with 9cRA partially blocked the loss of TH immunoreactivity in another 3 lesioned rats. (**B**) Labels in each panel correspond to the blocks at higher magnification in (**A**). Note that almost no TH cells in lesioned SN in the vehicle treated rat (**B**-**a**). In contrast, TH (+) cells were found in rostral (**B**-**b**) and caudal (**B**-**c**,**d**) SN in the lesioned rats treated with 9cRA.

**Figure 7:**
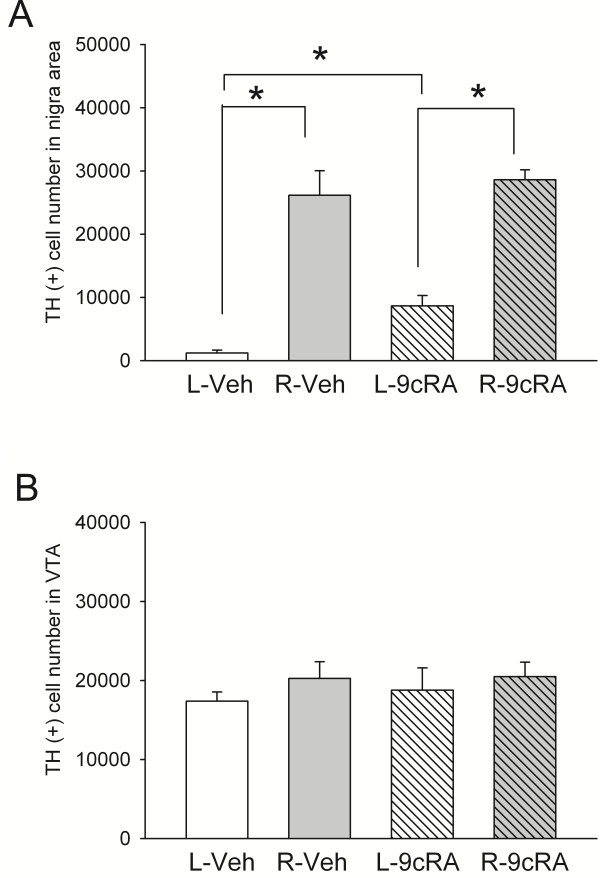
**9cRA treatment increased survival of TH cells in lesioned nigra. **TH (+) cell number (**A**) in SN and (**B**) in VTA were counted using unbiased stereology in 11 rats. (**A**) Injection of 6-OHDA reduced cell number of dopamine neurons in lesioned side (L) SN (p<0.001). Treatment with 9cRA significantly increased dopamine cell number in the lesioned side (L) SN (p=0.026). No change was found in the contralateral (non-lesioned, R) side SN (p=0.432). (**B**) TH cell number in A10. There is no significant difference after lesioning or after 9cRA treatment in VTA (p=0.734).

TH immunoreactivity was studied at >2 months after unilateral 6-OHDA lesioning, TH-positive [TH (+)] cell number in SN and in VTA were counted in 11 rats. Typical TH immunostaining of 6-OHDA -lesioned animals receiving either 9cRA or vehicle treatment is shown in Figure 6. Injection of 6-OHDA consistently reduced TH (+) cell number in SN in either vehicle or 9cRA –treated animals (p<0.001, 2-Way ANOVA; p<0.001, post-hoc Newman Keuls test, Figure 7A). Treatment with 9cRA significantly increased TH (+) cell number in the lesioned side SN (p=0.026, post-hoc Newman Keuls test) compared to vehicle. No change was found in the contralateral (non-lesioned) side SN (p=0.432). Although there is a trend that A10 TH cell number can be suppressed by 6-OHDA lesioning, there is no significant difference after lesioning or after 9cRA treatment (Figure 7B, p=0.734).

## Discussion

In this study, we found that density of TH (+) neurons and fibers in primary VM cultures were dose dependently reduced at 2 days after 6-hydroxydopamine treatment. 6-OHDA also induced DNA fragmentation as demonstrated in TUNEL labeling. These data are in agreement with our previous studies and suggest that administration of 6-OHDA causes activation of apoptotic pathways that lead to the death of VM dopaminergic neurons. We found that early and post treatment with 9cRA reduced 6-OHDA -mediated injury in VM cells. 6-OHDA -mediated reduction of TH cell density was significantly antagonized by 9cRA, suggesting that 9cRA is neuroprotective against 6-OHDA -mediated neurodegeneration in dopaminergic neurons *in vitro*.

Previous studies have demonstrated that RA exerts anti-apoptotic and antioxidant activity in neuronal and kidney cells. RA reduced staurosporine-induced oxidative stress and apoptosis by preventing the decrease in the levels of Cu-,Zn-superoxide dismutase (SOD-1) and Mn-superoxide dismutase (SOD-2) in primary hippocampal cultures [29] and by facilitating NGF-induced protection in chick embryonic neurons [30]. RA suppressed hydrogen peroxide –induced apoptotic nuclear condensation and membrane blebbing in rat glomeruli mesangial cells. Our recent studies also indicated that 9cRA reduced the density of TUNEL labeling, a marker for apoptosis/necrosis in ischemic cortex after middle cerebral artery occlusion in rats [14]. In this study, we demonstrated that treatment with 9cRA reduced 6-OHDA-mediated DNA fragmentation in culture. Taken together, our data suggest that 9cRA inhibits apoptosis and/or necrosis induced by 6-OHDA.

Using an established rodent model of PD, we also demonstrated the protective effect of 9cRA *in vivo*. Administration of amphetamine analogs causes ipislateral rotation in unilaterally 6-OHDA -lesioned rats due to differential increase of dopaminergic activity on the intact side. There is a correlation between amphetamine–induced ipislateral rotation and the depletion of dopamine in the nigra [31]. In this study, 9cRA was given after rotation experiment on day 6 post lesioning. We separated animals to 2 groups to balance rotational behavior before treatment; the difference in lesioning prior to treatment was thus minimal between two groups. We demonstrated that post-treatment with 9cRA, compared to vehicle, significantly attenuated rotation. Our data support that early and post-treatment with 9cRA reduced behavioral imbalance in hemiparkinsonian rats.

The protective effect of 9cRA is further supported by *in vivo* electrochemical data at 2 months after lesioning. We used high speed chronoamperometry to examine the time course of KCl -evoked DA release and clearance in striatum. The dose of KCl applied locally was between 7–14 × 10^-11^ mole (70 mM × 204.0 ± 9.1 nl) per site. The dose, concentration and volume of the KCl solution have been previously reported to induce depolarization and release of dopamine at dopaminergic nerve terminals *in vivo*[26,32]. We found that local administration of KCl induced DA release equally in the non-lesioned side striatum in 9cRA or vehicle –treated rats. In the lesioned side striatum, KCl-evoked DA release and the rate of DA clearance were greatly diminished in animals receiving vehicle treatment. Treatment with 9cRA did not induce a full recovery but significantly increased KCl-evoked DA release and DA clearance in the lesioned side striatum. These electrochemical data support that post-treatment with 9cRA protects against 6-OHDA –mediated deficiency in DA function in striatum.

Using unbiased stereology, we demonstrated that post-treatment with 9cRA significantly reduced the loss of TH cells in nigra. In contrast to a 95% reduction in TH cells in the lesion side nigra in animals with vehicle treatment, 70% TH (+) neurons were lost in the lesioned animals with 9cRA treatment. Similarly, there was about 99% reduction in KCl-evoked DA release in striatum in vehicle -treated animals. The reduction in DA release was only 66% after 9cRA treatment. Taken together, our data suggest that 9cRA has a protective effect against 6-OHDA injury in nigrostriatal dopaminergic neurons. It has been reported that the symptoms of the PD appear when 70-80% dopamine is depleted in patients [33,34]. In this study, we demonstrated that early treatment with 9cRA can reduce the death of DA neurons to 70%. It is thus possible that post-treatment with 9cRA can be beneficial to PD patients by slowing down its progressive neurodegeneration.

We previously demonstrated that 9cRA can selectively induce BMP7 mRNA expression in the brain. In this study, we demonstrated that 9cRA reduced 6-OHDA -mediated program cell death in culture. BMP7 has anti-apoptotic properties. BMPs reduced caspase-3 activation and DNA fragmentation in the ischemic brain [35,36] and attenuated dopaminergic neurotoxin -mediated apoptosis and cell death in nigrostriatal DA neurons. Interestingly, the reduction of TUNEL labeling by 9cRA in stroke brain was antagonized by the BMP antagonist noggin. Taken together, these data suggest that the protective effects of 9cRA may be indirectly mediated through a BMP signaling mechanism.

RA analogs, such as 9cRA and atRA, are agonists for RXR and RAR receptors. Compared to atRA, 9cRA is a more selective agonist for RXR. Although both atRA and 9cRA are neural protective against oxygen-glucose deprivation in hippocampal neurons [37], a differential sensitivity of these two ligands have been seen in other brain regions. Animals pretreated with 9cRA had lesser cortical infarction than those treated with atRA after middle cerebral artery occlusion [13]. Using RTPCR, we also found that 9cRA is more potent than atRA in inducing BMP7 (unpublished observation) and midkine [13] expression in primary cortical cultures. We demonstrated here that 9cRA is more potent than atRA, at 50 nM, against 6-OHDA –mediated neurodegeneration in TH neuronal culture. Taken together, these differential responses of 9cRA suggest that neuroprotection induced by 9cRA involves activation of RXR.

Although 9cRA has a high affinity for RXR, it is not detectable in adult brain tissue and, thus, may not be a candidate endogenous ligand for this receptor. Studies have indicated that the polyunsaturated fatty acids, such as linolenic and docosahexaenoic acid, activate RXR [10,38]. These polyunsaturated fatty acids have also been reported to reduce neurodegeneration [39,40]. Future experiments are required to investigate the mechanism of protection induced by these polyunsaturated fatty acids and their effects on RXR in animal model of PD.

There are several limitations to deliver drug to brain days after onset of 6-OHDA lesioning. Drugs given systemically may not easily cross the blood brain barrier (BBB) and can be degraded through first pass metabolism. Intracerebral delivery is not feasible for repeated drug administration and may require chronic cannulation. Previous reports have indicated that small molecules can by-pass the BBB and reach brain parenchyma non-invasively through an intranasal delivery. In this study, 9cRA was given initially through i.c.v. and then repeatedly through an intra-nasal route. We found that intranasal administration of 9cRA increased brain 9cRA level to 3 ng/g or 10 nM at 1 hour after delivery as detected by LC-MS/MS analysis (Additional file 1:Figure S1). No 9cRA was seen after vehicle treatment. Previous studies have shown that 9cRA can activate RXR receptor at this concentration [41].

In a preliminary study, we treated 6-OHDA –lesioned rats with intranasal 9cRA without i.c.v injection from day 7 to day 14. We did not find significant behavioral improvement after intranasal 9cRA treatment and days 20 and 30, suggesting that a loading dose of 9cRA, given i.c.v. on day 7 is required for this protective reaction.

## Conclusion

We have demonstrated that 9cRA, given from 7th day to 14th day post 6-OHDA lesioning, reduced rotational behavior and loss of TH cells in nigra, while increased DA release function in striatum. Our data suggest that 9cRA has neuroprotective effects against neurodegeneration in a rodent model of PD. The protection may be more prominent if 9cRA is given earlier or prior to lesioning. The effectiveness of 9cRA may be clinically useful for patients at early stages of PD.

## Methods

### Primary cultures of rat ventral mesencephalon

Primary cultures were prepared from embryonic (E14-15) ventral mesencephalon (VM) tissues obtained from fetuses of timed-pregnant Sprague–Dawley rats (Charles River Laboratories, Wilmington, MA), according to published procedures with some modification. The whole brain was removed aseptically and a small piece of tissue comprising the VM was dissected. After removing the blood vessels and meninges, pooled VM tissues were trypsinized (0.25%; Invitrogen, Carlsbad, CA) with gentle mixing for 15 min at 37°C. After rinsing off trypsin with pre-warmed DMEM/F-12 (Invitrogen), cells were dissociated by trituration, counted and plated into 96-well (6.0 × 10^4^/well) cell culture plates pre-coated with poly-lysine (Sigma-Aldrich). The culture plating medium consisted of Dulbecco’s modified Eagle medium/F12 supplemented with 10% heat-inactivated fetal bovine serum, 1 mM L-glutamine and 2% B27 (Invitrogen). Cultures were maintained at 37°C in a humidified atmosphere of 5% CO_2_ and 95% air. The cultures were fed by exchanging 50% of media with feed media (Neurobasal medium, Invitrogen) with 2% B27 with antioxidants (+AO) supplement on DIV (days *in vitro*) 3 and 5. ON DIV7, cultures were fed with feed media containing B27 supplement without antioxidants ((−) AO, Invitrogen). Freshly made 6-hydroxydopamine (in 20 μM ascorbic acid saline solution) or saline (with 20 μM ascorbic acid) was added to the wells on DIV 10. After incubation for 2 hours, cultures were washed with (−)AO B27 3 times. RA or vehicle was added to the well at the last wash. Cells were returned to a 37°C incubator for 22 hours then fixed with 4% paraformaldehyde (PFA) for immunoreactivity.

### *In vitro* immunoreactivity and quantitation

After removing PFA solution, cells were washed with PBS and the fixed cultures were treated for 1 hour with blocking solution (2% BSA, 0. 1% Triton X-100 and 5% goat serum in PBS). The cells were then incubated for 2 days at 4°C with a mouse monoclonal antibody against TH (1:500; Chemicon, Temecula, CA, USA). The cells were then rinsed three times in PBS. The bound primary antibody was visualized using the AlexaFluor 488 goat anti-mouse secondary (Invitrogen). Images were acquired using a SPOT RT camera (Diagnostic Instruments, Inc., Sterling Heights, MI) attached to a NIKON TE2000 inverted microscope. TH+ cells were manually counted in 10x images (4 fields per well of 96 well plate). All immunoreactive counts and quantitation were expressed as percentage of untreated cells. Experiments were repeated 2–3 times with n=3-9 wells per group per experiment.

### *In vitro* Terminal deoxynucleotidyl transferase (TdT)-mediated dNTP nick -end labeling (TUNEL)

Cultures were assayed for DNA fragmentation using a TUNEL-based method (In Situ Cell Death Detection Kit; Roche, Indianapolis, IN). Briefly, 4% PFA fixed cells were permeabilized in 0.1% Triton X-100 in 0.1% sodium citrate for 2 min on ice. To label damaged nuclei, 50 μL of the TUNEL reaction mixture was added to each sample and kept at 37 °C in a humidified chamber for 60 min. Procedures for positive and negative controls were carried out as described in the manufacturer’s manual (Roche). Controls consisted of not adding the label solution (terminal deoxynucleotidyl transferase) to the TUNEL reaction mixture. Material was examined using a Nikon TE2000 inverted microscope equipped with fluorescence. TUNEL(+) cells were manually counted in 20x images (4 fields per well of 96 well plate).

### Animals

Adult male Sprague Dawley rats from Charles River Lab Inc. were used for this study. The use of animals was approved by the Animal Care and Use Committee, National Institute on Drug Abuse, IRP.

### 6-hydroxydopamine lesioning

Rats were anesthetized with chloral hydrate (400 mg/kg, i.p.) and placed in a stereotaxic frame. 6-hydroxydopamine (2.27 μg/μl x 5 μl in 0.9% NaCl containing 0.2 mg/ml ascorbic acid) was unilaterally injected into the left medial forebrain bundle (−4.4 mm AP, 1.2 mm ML relative to bregma and 8.4 mm below skull) over 4 min. After injection, a piece of bone wax (W810, Ethicon) was applied to the burr hole in the skull to prevent efflux of the solution.

### Behavioral measurement

Rotational behavior [42,43] was evaluated using a multichannel rotometer system (RotoMax, AccuScan Instruments, Inc). Six days after 6-OHDA lesioning, all animals were challenged with methamphetamine (2.5 mg/kg). Each animal was placed in a cylindrical test chamber for 90 min. The highest consecutive net rotations (ipsilateral -contralateral rotation) over 60 min were used for analysis. Animals that rotated in excess of 300 turns/hour were selected for 9cRA or vehicle treatment. Methamphetamine –induced rotation was re-examined on 20 and 30 days after lesioning.

### Administration of 9cRA

Animals were separated into 2 groups to receive 9cRA or vehicle after rotation was measured by rotometer on day 6 after 6-OHDA lesioning. On the 7^th^ day after lesioning, animals were anesthetized with chloral hydrate (0.4 g/kg, i.p.). 9cRA (1 μg/1μl × 20 μl, dissolved in 10% DMSO, Sigma, pH 7.0) or vehicle (10% DMSO in saline pH 7.0, 20 μL) was given intracerebroventricularly through a Hamilton syringe. The coordinates for intracerebroventricular injections were: 0.8 mm posterior to the bregma, 1.5 mm lateral to the midline; 3.5 mm below dura surface. The needle was retained in place for 5 min after injection. After injection, a piece of bone wax (W810, Ethicon) was applied to the burr hole in the skull to prevent efflux of the solution. Animals were anesthetized with isoflurane for 5–10 min each day from day 8 to day 14 after lesioning and were placed in a supine position. 9cRA (conc=1 μg/1μl) or vehicle was delivered into nostrils of each rat at dose of 20 μl daily as previously described [44]. No difference in body weight was found between animals treated with 9cRA or vehicle.

### KCl-evoked DA release

KCl –evoked DA release in striatum was measured at 2 months after lesioning. Animals were anesthetized using urethane (1.25 g/kg, i.p.). In-vivo chronoamperometric measurements of extracellular dopamine (DA) concentration were performed as previously described (Zhou et al., 1996). The recordings were taken at rates of 10 Hz continuously using Nafion-coated carbon-fiber working electrodes (tip = 30 μm; SF1A, Quanteon, Lexington, Kentucky) and a microcomputer-controlled apparatus (FAST system, Quanteon). The release of DA was measured by changes in extracellular DA concentration after microinjection of KCl into the striatal parenchyma. KCl (70 mM) in osmolarity balanced saline (79 mM NaCl and 2 mM CaCl2) was locally applied through a micropipette in the 150 to 225 nl range. The concentration and volume of KCl solution in the pipette have been previously reported to induce depolarization and release of dopamine at dopaminergic nerve terminals *in vivo*. The working electrode and the micropipette were mounted together with sticky wax; tips were separated by 150 μm. The electrode/pipette assembly was lowered into striatum (AP 0–0.5 mm, M/L 2.5 mm relative to bregma and 3.5 to 7.0 mm below the dura). Local application of KCl from the micropipette was performed by pressure ejection using a pneumatic pump (BH2, Medical System). The ejected volume was monitored by recording the change in the fluid meniscus in the pipette before and after ejection using a dissection microscope.

### Tyrosine hydroxylase (TH) immunoreactivity

Animals were euthanized >2 month after lesioning. Animals were anesthetized with chloral hydrate (400 mg/kg i.p.) and perfused transcardially with saline followed by 4% paraformaldehyde (PFA) in phosphate buffer (PB; 0.1 M; pH 7.2). The brains were dissected, post-fixed in PFA for 18–20 hours, and transferred to 18% sucrose in 0.1 M PB for at least 16 hours. Serial sections of the entire brain were cut at 40 μm thickness in a cryostat. One series from every sixth section was stained for TH. In order to control for staining variability, specimens from all experimental groups were included in every batch and reacted together in a net well tray under the same conditions. Sections were rinsed in 0.1M phosphate buffer (PB), blocked with 4% bovine serum albumin (BSA) and 0.3% Triton x-100 in 0.1M PB. Sections were then incubated in a primary antibody solution mouse monoclonal anti-TH diluted in 4% BSA and 0.3% Triton x-100 in 0.1M PB, concentration 1:100 (Chemicon, Temecula, CA) for 17–19 hours at 4°C. Sections were then rinsed in 0.1M PB and incubated in biotinylated horse anti-mouse IgG in the buffer (1:200; Vector Laboratories, Burlingame CA) for 1 hour, followed by incubation for 1 hour with avidin-biotin-horseradish peroxidase complex. Staining was developed with 2,3′ diaminobenzidine tetrahydrochloride (0.5 mg/ml in 50 mM Tris–HCl buffer 7.4). Control sections were incubated without primary antibody. Sections were mounted on slides, and cover slipped.

### Stereological analysis

The total number of TH (+) neurons was estimated bilaterally every 6^th^ section through the extent of the midbrain for adult control and 6-OHDA treated rats. The substantia nigra compacta (SNc) was outlined under a low magnification objective (5x) following landmarks from the Paxinos and Watson rat atlas (Paxinos, G, 2004) and the stereologic analysis was performed under the 40x objective of a Leica DM5000B microscope (Leica Microsystems, Bannockburn, IL) and analyzed with StereoInvestigator software (MBF Bioscience, Williston, VT). The optical fractionator probe was used to generate an estimate of the total number of TH (+) neurons in the SNc. For each tissue section analyzed, section thickness was assessed in each sampling site and guard zones of 2.5 μm were used. Systematic random sampling design was performed and generated with the following stereologic parameters: grid size: 270 × 185μm, counting frame: 100 × 100μm and dissector height of 25 μm. Our criterion for counting an individual TH (+) neuron was the presence of its nucleus. Coefficients of error were calculated and values <0.10 were accepted.

## Abbreviations

6-OHDA: 6-hydroxydopamine; 9cRA: 9 cis retinoic acid; (−)AO: Without antioxidants; (+)AO: With antioxidants; atRA: All-trans retinoic acid; BBB: Blood brain barrier; BMP-7: Bone morphogenetic protein-7; DA: Dopamine; GDNF: Glial cell line derived neurotrophic factor; MPP+: 1-methyl-4-phenylpyridinium; PD: Parkinson’s disease; PFA: Paraformaldehyde; RA: Retinoic acid; RAR: Retinoic acid receptor; RXR: Retinoid X receptor; SOD: Superoxide dismutase; TGF: Transforming growth factor; TH: Tyrosine hydroxylase; TUNEL: Terminal deoxynucleotidyl transferase dUTP nick end labeling; VM: Ventromesenphalic.

## Competing interests

The authors declare that they have no competing interests.

## Authors’ contributions

LY and HS carried out the surgery, behavioral and electrochemical studies. OD, CB, SY and YW carried out immunohistochemical studies. EB carried out neuronal culture study. SY and YW drafted the manuscript. All authors read and approved the final manuscript.

## Supplementary Material

Additional file 1 **Figure S1.** Increase 9cRA level in brain after intranasal delivery of 9cRA. A total of 12 rats were given either 9cRA (20 ug per animal) or vehicle intranasally. Brain tissues were harvested at 1, 2 and 4 hours after administration. Tissues were extracted and 9cRA levels in brain homogenates were detected by Mass spectrometry as previously described (Kane, A. Biochem J. 388,363-369, 2005). No detectable 9cRA was found in vehicle treated rats. There is a significant increase in 9cRA level at one and 2 hours after 9cRA administration (p<0.05, one way ANOVA). Brain 9cRA level returned to the basal level at 4 hours after administration. Click here for file
