# Supplementary figures and images for: Early post-treatment with 9-cis retinoic acid reduces neurodegeneration of dopaminergic neurons in a rat model of Parkinson’s disease
Source: BMC Neurosci. 2012 Oct 6;13:120. doi: 10.1186/1471-2202-13-120 (PMC3523975; doi:10.1186/1471-2202-13-120)

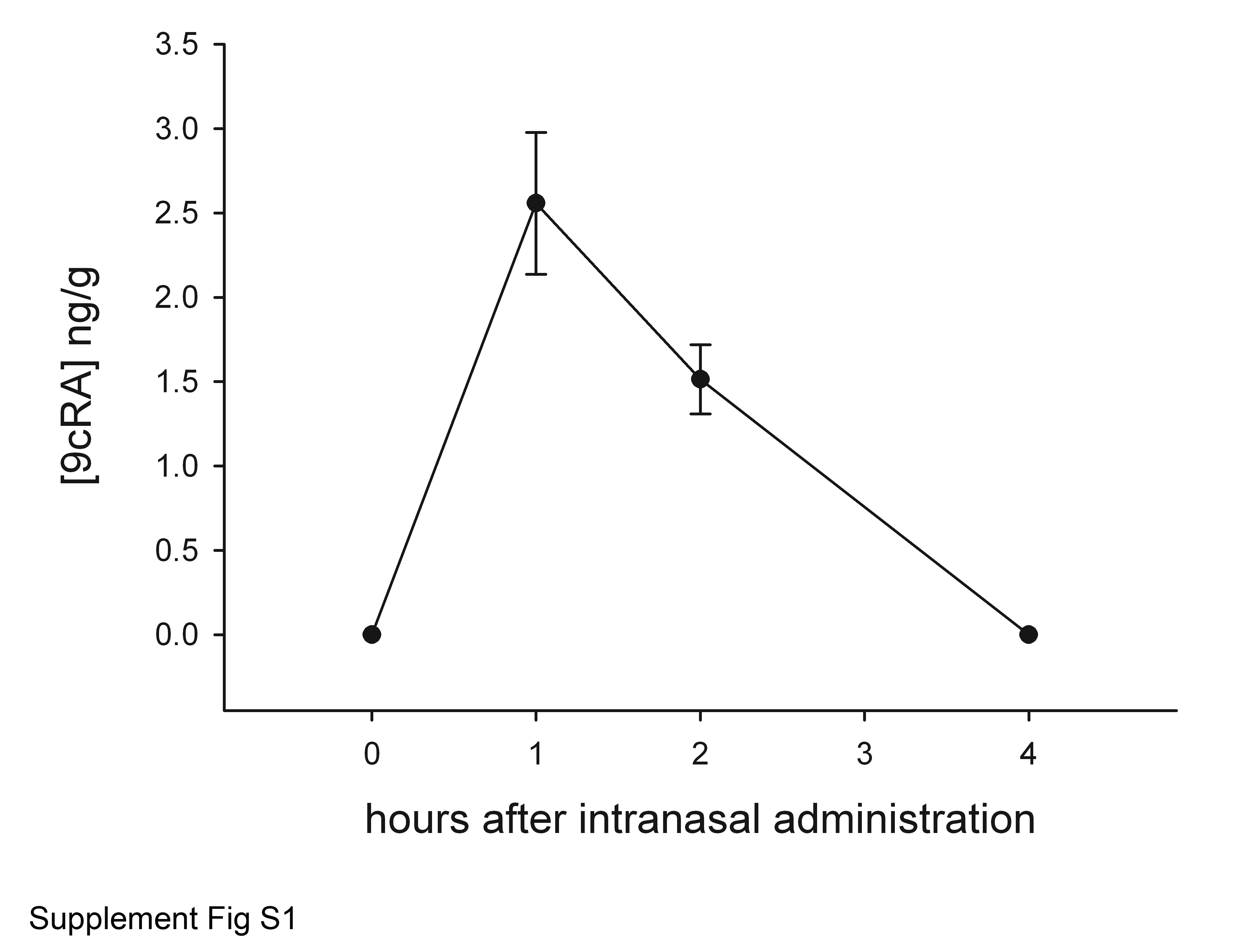

Supplement: Additional file 1 — Figure S1. Increase 9cRA level in brain after intranasal delivery of 9cRA. A total of 12 rats were given either 9cRA (20 ug per animal) or vehicle intranasally. Brain tissues were harvested at 1, 2 and 4 hours after administration. Tissues were extracted and 9cRA levels in brain homogenates were detected by Mass spectrometry as previously described (Kane, A. Biochem J. 388,363-369, 2005). No detectable 9cRA was found in vehicle treated rats. There is a significant increase in 9cRA level at one and 2 hours after 9cRA administration (p<0.05, one way ANOVA). Brain 9cRA level returned to the basal level at 4 hours after administration. [file 1471-2202-13-120-S1.tiff]
